# Comparing detectability patterns of bird species using multi-method occupancy modelling

**DOI:** 10.1038/s41598-021-81605-w

**Published:** 2021-01-28

**Authors:** José M. Zamora-Marín, Antonio Zamora-López, José F. Calvo, Francisco J. Oliva-Paterna

**Affiliations:** 1 Departamento de Zoología y Antropología Física, Facultad de Biología, Universidad de Murcia, Murcia, Spain; 2 Departamento de Ecología e Hidrología, Facultad de Biología, Universidad de Murcia, Murcia, Spain

**Keywords:** Behavioural ecology, Community ecology, Ecological modelling

## Abstract

A robust knowledge of biodiversity distribution is essential for designing and developing effective conservation actions. The choice of a suitable sampling method is key to obtaining sufficiently accurate information of species distribution and consequently to improve biodiversity conservation. This study applies multi-method occupancy models to 36 common bird species associated with small ponds in the province of Murcia (south-eastern Spain), one of the most arid regions of Europe, in order to compare their effectiveness for detecting different bird species: direct observation, combined observation and video monitoring and mist netting captures. The results showed that the combined method and direct observation were similar and most effective than mist netting for detecting species occupancy, although detection rates ranged widely among bird groups, while some large species were poorly detected by all the methods used. Average detectability did not increase during the breeding period. The chosen approach is particularly applicable to both single- and multi-species bird monitoring programmes. However, we recommend evaluating the cost-effectiveness of all the available methods in order to reduce costs and improve the success of sampling designs.

## Introduction

Monitoring biodiversity is key to assessing the status and trends of wildlife as well as for understanding its response to threats derived from human activities. Species richness and abundance are the most widely used biological measurements in ecological studies and are frequently provided by large-scale monitoring programmes^1,2^. However, despite their importance for biodiversity management and conservation, most programmes are under-resourced^3^, placing constraints on the number of target species, sampling effort and kind of sampling methods used to detect the target species chosen^4^. Such limitations in survey design may well contribute to large biases in detection probabilities, leading to the misinterpretation of abundance and distribution estimates. Indeed, concern about bias in species detectability has historically been expressed by ecologists, but the interest in incorporating imperfect detection into ecological studies is relatively recent^5,6^ and has largely increased in the last two decades due to the development of hierarchical modelling techniques^7,8^. For example, some studies have reported extremely inaccurate richness estimates as a result of not taking into account possible imperfect detection, masking trends and providing misinformation that can affect conservation actions^9,10^. Hence, setting an accurate study design based on effective sampling methods that maximize species detectability is a key factor in any biological monitoring programme.

The probability of detection or detectability (*p*) is defined as the probability of detecting at least one individual of a given species in a single site during a survey, given that individuals of that species are present in that site during the sampling period^5,11^. Traditionally, the vast majority of studies have assumed all the species composing a biological community are similarly detected^7^, and detectability is constant over space and time despite the different methods used or weather conditions. The hierarchical modelling framework allows different approaches to be considered in order to estimate distribution, abundance and species richness corrected for imperfect detection^2,12^. For example, single-species occupancy models can be applied to the data of presence-absence surveys in order to map predicted distributions or to understand species-specific detectability^13^. On the other hand, multi-species occupancy models enable unbiased estimates of site-specific species richness to be calculated while accounting for imperfect detection^8^, thus enhancing richness predictions in studies that tended only to use observed richness^10^. Furthermore, many of these models also allow the incorporation of covariate relations in order to explore the influence of biotic and abiotic factors on species richness or the distribution or abundance of target species^2,14^.

The simplest occupancy models accounting for imperfect detection entail two different processes: an ecological process governed by the probability of occupancy and another observation process that is governed by the probability of detection^1,2^. The former is defined by the species requirements (habitat, geographical range and climate) and depends on the true occupancy state, involving both the presence and distribution of target species in the study area (i.e. whether the species is or is not present). The latter process depends directly on occupancy and is governed by the same drivers (i.e. whether the target species is or is not detected). A species can only be detected in a sampling unit survey when that species is occupying the study unit. Besides drivers of occupancy, assuming population closure^1^, the observation process is constrained by several additional factors that hinder or modulate the detectability of species. These factors are derived firstly from species-specific traits, such as behaviour, life history and phylogenetic relatedness^15,16^, and secondly from study design features, such as time of survey^17^, sampling method, survey effort (number of surveys and sampling units), weather conditions, surveyor skills and habitat characteristics among others^13,16^. Presence-absence data across several surveys of the sampling units are required to estimate the probability of detection for any species. However, some different extensions have recently been applied to single-visit datasets in order to deal with this constraint; for example, it is possible to account for multiple independent observers, multiple independent detection methods (multi-method) or by the spatial subsampling of the study area^13,18,19^.

Currently, birds are the most frequently used group for occupancy modelling, probably due to the greater number of datasets and statistical methods available^7^. To date, most bird studies have accounted for imperfect detection by using data from visual and aural point counts^15,17,20,21^. However, a similar effectiveness for detecting species richness has been reported for mist netting^22,23^, a sampling method based on trapping birds with nets in order to mark them individually, a technique that has been increasingly used over recent decades^24^. There is a large literature contrasting both sampling methodologies based on descriptive approaches in terms of richness and abundance^25–28^. For example, Rappole et al.^22^ used data from point counts and mist netting in tropical habitats to show different method-specific biases and proposed a combined methodology to provide a more accurate assessment of the avian community. Despite similar effectiveness in detecting species richness, most of these studies have pointed to the greater bias of mist netting when recording the abundance of bird species^23,29^.

On the other hand, the rapid development of new technologies is revolutionizing biodiversity monitoring, and several devices can now be used to record large amounts of field data^3,30^. For example, video cameras have recently been used to explore drinking patterns of desert birds in small manmade ponds in areas of Arizona and Kalahari^31,32^. In arid and semi-arid regions, artificial water bodies such as drinking troughs and cattle ponds may represent the only drinking water sources for ensuring terrestrial biodiversity^31^, thus providing a key service for wildlife. Therefore, these aquatic systems act as an ideal model habitat for detecting biodiversity and exploring detectability patterns in areas with scarce water availability.

Over the last years, an increasing number of studies have explored the effectiveness of different sampling methods through a multi-method modelling approach, most of them focusing on mammal species^33–35^. Here, we use multi-method occupancy models^35^ to compare the effectiveness of three sampling methods for detecting 36 breeding bird species. For that purpose, 19 isolated small ponds located in a semi-arid region were selected as model habitat for the three sampling techniques to be applied. Detectability estimates were calculated for each method at species level. Our specific aims were to: (1) compare the detection effectiveness of different sampling methods in breeding bird species; (2) assess the contribution of sampling date as a source of variation in detection probabilities during the breeding season and; (3) explore the influence of phylogenetic relatedness and life-history traits on species detectability at method level. The multi-method occupancy modelling carried out could be used as a starting point in the design stage of biological monitoring programmes, allowing resource optimization and maximizing the detectability of target species.

## Results

**Figure 1:**
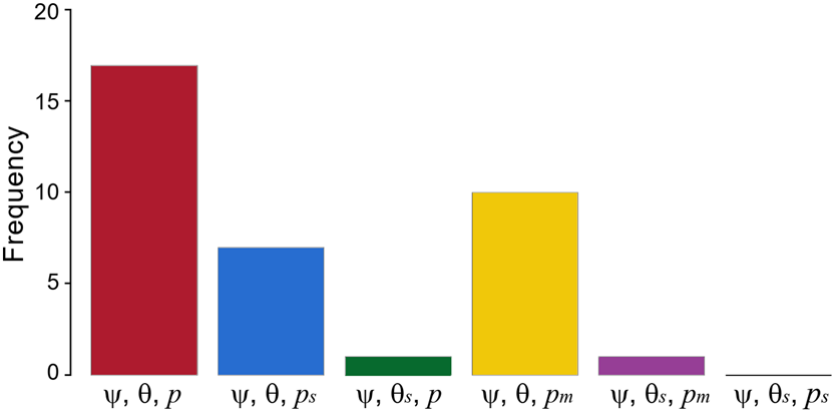
Frequency of best models explaining detection estimates of 36 bird species recorded in ponds in south-eastern Spain. For each species, six candidate models were considered (see descriptions in Table 2). The selection procedure for each species was based on the lowest AICc value. The figure was created in R (version 4.0.2, https://www.R-project.org/) and assembled with GIMP (version 2.10.14, https://www.gimp.org/).

A total of 5304 birds belonging to 36 species recorded in small ponds during the sampling season were used to model occupancy and detectability (Table 1). Another 26 taxa belonging to migratory non-breeding birds in the study area, such as the Pied Flycatcher (*Ficedula hypoleuca*) and Willow Warbler (*Phylloscopus trochilus*), and occasional species with less than five records were removed from the statistical analysis. The results revealed that the null model was the best supported model for 47.2% of bird species (17 taxa), followed by the method-specific model and survey-specific model for 27.8% (10 taxa) and 19.4% (7 taxa) of the species, respectively (Fig. 1, Supplementary Tables S1 and S2). The models considering survey-dependent availability effects (θ_*s*_) were largely unsupported for most species.

**Table 1:** Summary of species name, family and group membership for breeding bird species recorded in pond surveys in south-eastern Spain.

Species	Common name	Family	Bird group
*Columba palumbus*	Common Woodpigeon	Columbidae	5
*Streptopelia turtur*	European Turtle-dove	Columbidae	5
*Turdus viscivorus*	Mistle Thrush	Turdidae	2
*Turdus merula*	Eurasian Blackbird	Turdidae	2
*Luscinia megarhynchos*	Common Nightingale	Muscicapidae	3
*Erithacus rubecula*	European Robin	Muscicapidae	1
*Phoenicurus ochruros*	Black Redstart	Muscicapidae	1
*Saxicola torquata*	Common Stonechat	Muscicapidae	1
*Muscicapa striata*	Spotted Flycatcher	Muscicapidae	3
*Hippolais polyglotta*	Melodius Warbler	Acrocephalidae	3
*Phylloscopus collybita*	Common Chiffchaff	Phylloscopidae	1
*Phylloscopus bonelli*	Western Bonelli's Warbler	Phylloscopidae	1
*Sylvia hortensis*	Western Orphean Warbler	Sylviidae	3
*Sylvia undata*	Dartford Warbler	Sylviidae	3
*Sylvia cantillans*	Subalpine Warbler	Sylviidae	3
*Sylvia melanocephala*	Sardinian Warbler	Sylviidae	3
*Periparus ater*	Coal Tit	Paridae	1
*Lophophanes cristatus*	Crested Tit	Paridae	1
*Parus major*	Great Tit	Paridae	1
*Cyanistes caeruleus*	Eurasian Blue Tit	Paridae	1
*Aegithalos caudatus*	Long-tailed Tit	Aegithalidae	1
*Sitta europaea*	Eurasian Nuthatch	Sittidae	3
*Certhia brachydactyla*	Short-toed Treecreeper	Certhiidae	1
*Lanius senator*	Woodchat Shrike	Laniidae	2
*Garrulus glandarius*	Eurasian Jay	Corvidae	6
*Pica pica*	Eurasian Magpie	Corvidae	6
*Petronia petronia*	Rock Sparrow	Passeridae	6
*Fringilla coelebs*	Common Chaffinch	Fringillidae	4
*Serinus serinus*	European Serin	Fringillidae	4
*Carduelis chloris*	European Greenfinch	Fringillidae	3
*Carduelis carduelis*	European Goldfinch	Fringillidae	4
*Carduelis cannabina*	Common Linnet	Fringillidae	4
*Loxia curvirostra*	Red Crossbill	Fringillidae	5
*Emberiza calandra*	Corn Bunting	Emberizidae	5
*Emberiza cia*	Rock Bunting	Emberizidae	4
*Emberiza cirlus*	Cirl Bunting	Emberizidae	4

Bird groups were established based on body size and main diet type: (1) small insectivorous (< 30 g); (2) medium-sized and large insectivorous (≥ 30 g); (3) small insectivorous and frugivorous (< 30 g); (4) small seed-eaters (< 30 g); (5) medium-sized and large seed-eaters (≥ 30 g); and (6) medium-sized and large generalists (≥ 30 g).

**Figure 2:**
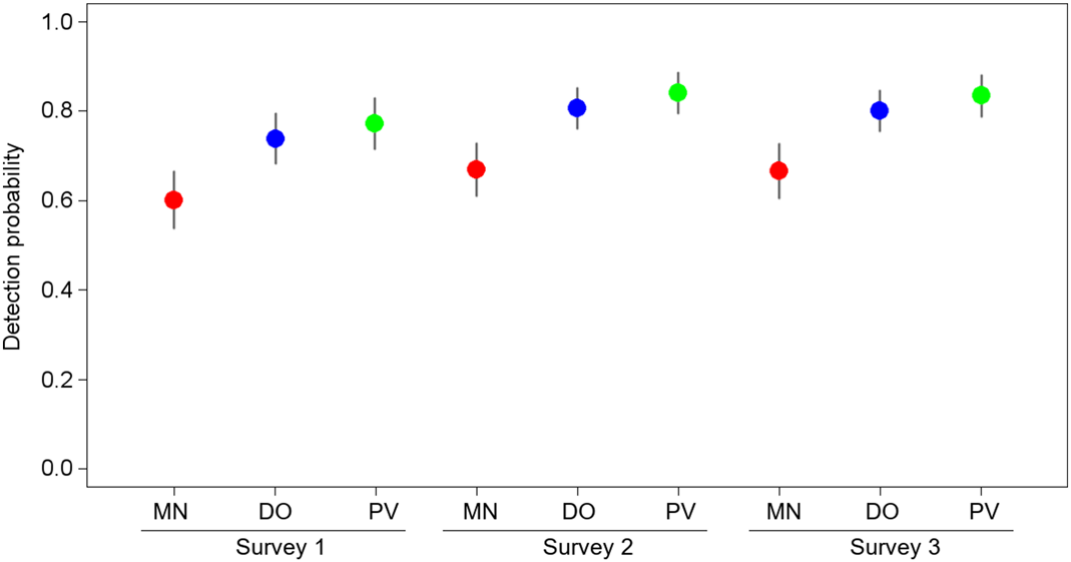
Model-averaged detection estimates of 36 bird species recorded in pond surveys in south-eastern Spain. Probabilities are averaged by sampling method and survey occasion. Vertical lines represent 95% confidence intervals. The three sampling methods are indicated: *MN* mist netting, *DO* direct observation, *PV* direct observation plus video monitoring. Surveys 1, 2 and 3 correspond to visits made in early-mid spring, late spring and early summer, respectively. The figure was created in R (version 4.0.2, https://www.R-project.org/) and assembled with GIMP (version 2.10.14, https://www.gimp.org/).

Model-averaged estimates of species detection probabilities showed differences depending on the sampling method (Fig. 2). Occupancy detection increased very slightly during the breeding season but the pattern of differences among the three sampling methods remained similar for all three surveys. Direct observation (DO) and direct observation plus video monitoring (PV) provided similar detectability estimates. PV provided detectability estimates substantially higher than mist netting captures (MN), but the other pairwise comparisons did not point to any relevant differences. Nevertheless, detection estimates of some species were low even in the case of PV. MN provided the lowest detectability estimates of the three studied methods. Moreover, MN showed the highest variability in species detectability because this method covered a wide range from almost full detection (*p* = 1) for some species (e.g. *Carduelis chloris* and *Serinus serinus*) to practically null detection (*p* = 0) for others such as *Columba palumbus* (Supplementary Table S3).

**Figure 3:**
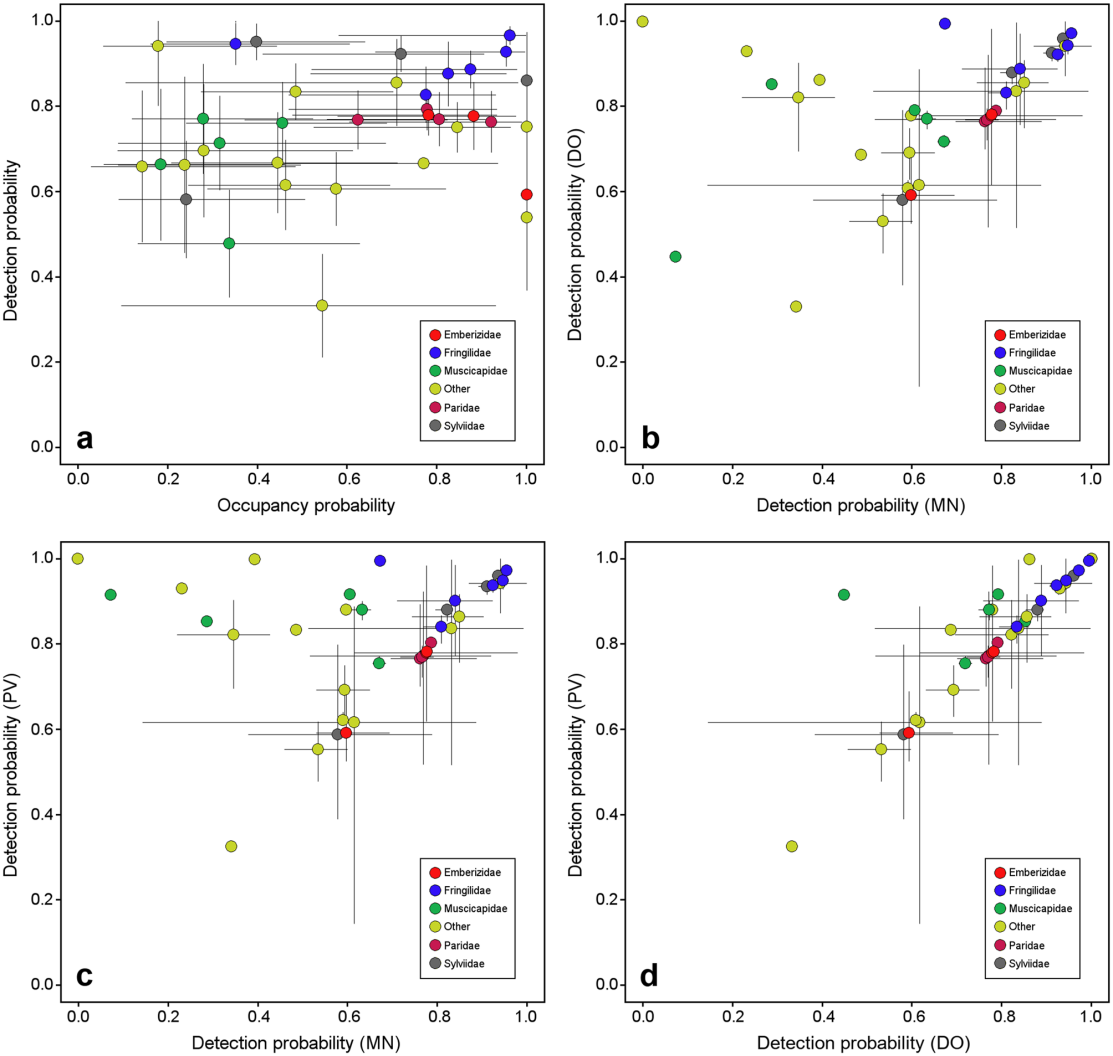
Occupancy and detection probabilities of 36 bird species recorded in pond surveys in south-eastern Spain. (**a**) Occupancy versus detection probabilities. Occupancy probabilities are model-averaged occupancy estimates (ψ). Detection probabilities are the mean of the nine method- and survey-specific, model-averaged detection estimates. Lines represent 95% confidence intervals. (**b–d**) Pairwise comparison of detection probabilities for mist netting (MN), direct observation (DO) and direct observation plus video monitoring (PV). Points represent the average of the three survey-specific estimates and lines represent the range. Point colour refers to avian family. Only the five families with the highest number of recorded species are indicated, while the remaining families are grouped as “other”. The figure was created in R (version 4.0.2, https://www.R-project.org/) and assembled with GIMP (version 2.10.14, https://www.gimp.org/).

The occupancy estimates ranged widely from ψ = 0.14 (95% CI: 0.03, 0.48) in *Sitta europaea* to ψ = 1 (95% CI: 1.00, 1.00) in *Turdus merula*. However, the detection estimates for many studied species (86.1%) was higher than *p* = 0.6, and only five of the 36 modelled species showed lower values (Fig. 3a). It should be noted that two of these five species, *Phylloscopus collybita* and *Emberiza calandra*, exhibited a relatively low average detectability (*p* < 0.6) although their occupancy was complete (ψ = 1; 95% CI 1.00, 1.00). The family with the highest occupancy and detection estimates were finches (Fringillidae), all species of which showed ψ > 0.77 and *p* > 0.82, except *Carduelis chloris*, which had a low estimated occupancy value (ψ = 0.35; 95% CI 0.16, 0.60). The availability estimates (θ_*s*_) ranged widely variable across species but were relatively constant across surveys (Supplementary Table S1).

Approximately half of the 36 bird species recorded were similarly detected by the three methods (Fig. 3b–d). However, both observation methods (DO and PV) were much more effective than MN for detecting species such as *Columba palumbus*, *Pica pica*, *Muscicapa striata*, *Streptopelia turtur* and *Garrulus glandarius*. Otherwise, MN was no more effective than observational methods for any of the species modelled, except for *Certhia brachydactyla* whose estimate was very slightly higher with MN (Supplementary Table S3).

**Figure 4:**
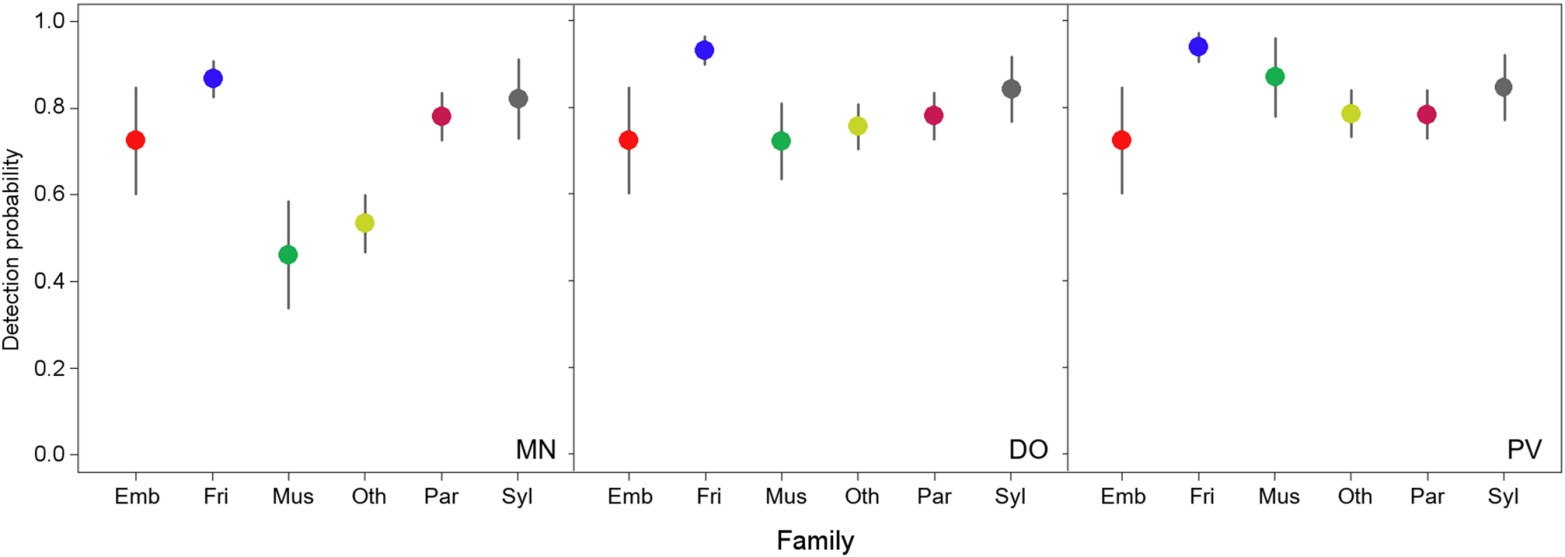
Bird species detectability at family level for each of the three methods deployed in pond surveys in south-eastern Spain. Method-specific, model-averaged estimates of detection probabilities (independent of the survey time) grouped by species family. Vertical lines represent 95% confidence intervals. Only the five families with the highest number of recorded species are indicated, the remaining families being grouped as “other”. Families are indicated as follows: *Emb* Emberizidae, *Fri* Fringillidae, *Mus* Muscicapidae, *Oth* other families, *Par* Paridae, and *Syl* Sylviidae. Survey method label appears in the bottom right corner as follows: *MN* mist netting, *DO* direct observation, and *PV* direct observation plus video monitoring. The figure was created in R (version 4.0.2, https://www.R-project.org/) and assembled with GIMP (version 2.10.14, https://www.gimp.org/).

**Figure 5:**
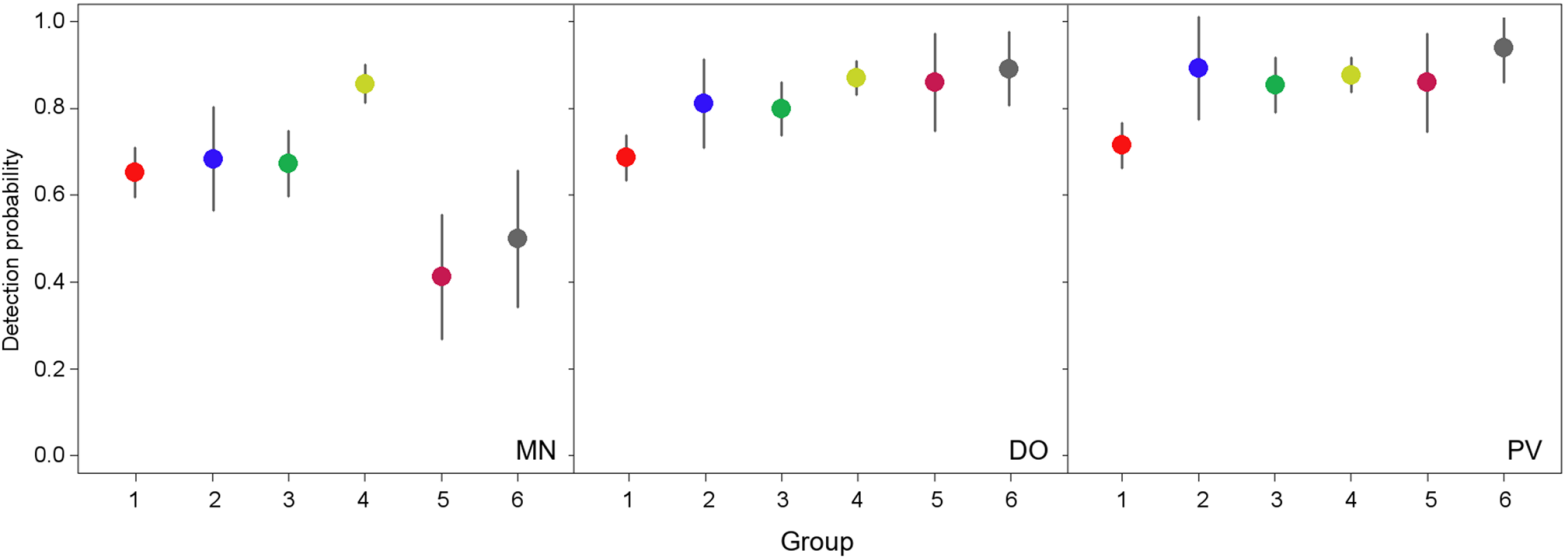
Bird species detectability at group level for each of the three methods deployed in pond surveys in south-eastern Spain. Method-specific, model-averaged estimates of detection probabilities (independent of the survey time) grouped by species group. Vertical lines represent 95% confidence intervals. Numbers refer to six different established groups based on body size and main diet type: (1) small insectivorous (< 30 g); (2) medium-sized and large insectivorous (≥ 30 g); (3) small insectivorous and frugivorous (< 30 g); (4) small seed-eaters (< 30 g); (5) medium-sized and large seed-eaters (≥ 30 g); and (6) medium-sized and large generalists (≥ 30 g). Sampling method label appears in the bottom right corner as follows: *MN* mist netting, *DO* direct observation, and *PV* direct observation plus video monitoring. The figure was created in R (version 4.0.2, https://www.R-project.org/) and assembled with GIMP (version 2.10.14, https://www.gimp.org/).

Six of the 36 modelled species were only recorded by observation methods. These corresponded to large birds (such as *Streptopelia turtur*, *Pica pica* and *Columba palumbus*) or species with few records (*n* < 10, such as *Luscinia megarhynchos* and *Lanius senator*). However, no species were detected by MN alone. Contrasting results at family level were found in the case of method-specific detectability (Fig. 4). Observational methods showed substantially higher effectiveness than MN for detecting the Muscicapidae family (flycatchers), the families grouped as Other and, to a lesser extent, the Fringillidae family (finches). However, the estimated detectability of the rest of families was similar for the three sampling methods. Detectability with the DO and PV methods was very similar for all the studied families, except the Muscicapidae family which were slightly better detected by PV. On the other hand, visual methods in general were also more effective than MN at detecting species at group-level (Fig. 5). Detection probability for small insectivore and frugivore species (group 3) increased slightly from MN to DO and PV, whereas small insectivorous, medium-sized and large insectivorous and small seed-eaters (groups 1, 2 and 4, respectively) showed similar detection probability among the three survey methods. Moreover, visual methods were more effective than MN at detecting medium-sized and large seed-eaters and generalists (groups 5 and 6). Detectability by DO and PV was very similar for all bird groups.

Detectability over the whole survey period was very similar for almost all the avian families and groups (Supplementary Figs. S1 and S2). Survey-specific detection estimates for each of the three sampling methods are reported in Supplementary Table S3.

## Discussion

Multi-method occupancy models can be used to compare effectiveness among different sampling techniques for monitoring wildlife. In this study, we used an occupancy modelling approach to assess imperfect detection in bird species surveys based on three different sampling methods. This approach allowed us to calculate both method-specific and survey-specific detection estimates for 36 breeding bird species associated with small ponds, which represent 30.0% of the terrestrial breeding bird community in the study area^36^.

Visual methods (DO and PV) were more effective for occupancy detection than MN. Unsurprisingly the detection estimates for both visual methods were very similar, suggesting that the additional use of video cameras does not provide apparent improvement over the results obtained by the most traditional method of DO. However, the additional use of video cameras may be regarded as a useful monitoring tool in biodiversity studies in habitats that have a pull-effect on birds, such as ponds or animal feeders^31,37^, although this effect is appropriately dealt with the multi-method approach^35^. Because the multi-method model estimates incomplete availability, its use is appropriate for habitats with a pull effect that would violate the closure assumption for the standard single-season occupancy model, and therefore provides valid estimates of detection when changes in occupancy may occur between sampling intervals.

The detectability averaged over the three methods showed similar estimates for closely related species. Warblers (Sylviidae) and finches were the avian families with highest detectability, with eight species having a probability of detection ranging from 0.85 to 0.96. In this respect, phylogenetic relatedness has been reported as a driver of species detectability so that closely related taxa are expected to show similar detection rates^15^. Moreover, six of these eight species were the most abundant bird species in our study, suggesting avian abundance influences the detection process, as reported in previous studies^13^. On the other hand, the detectability of flycatchers showed significant differences between sampling methods, PV being the best method for recording these species, closely followed by DO. The higher effectiveness of visual techniques to detect flycatchers is probably explained by their conspicuous feeding behaviour, which makes them easily detectable.

Previous studies have pointed to the influence of survey date on bird detectability. For example, several species show unchanged detectability with time, whereas others show strongly increasing or sharply decreasing time-dependent detectability^17^. An increase in population abundance may be responsible of an increase in detectability^38^, which can be explained by the incorporation of fledgling birds to the population during the breeding season. Moreover, in arid zones, birds have been reported to use water bodies more frequently during hot periods^31,39^, with abundance and species detectability increasing as a consequence. However, for most species, models considering survey-dependent availability had very low support from the data, and our results showed no general increase in detection probability as the breeding season progressed with all three sampling methods.

Mist netting was ineffective at detecting both medium-sized and large seed-eaters and generalist birds, such as doves and crows, or species with a very patchy distribution in the study area, such as nightingales and flycatchers. However, MN was effective at recording the presence of two small warbler species (*Sylvia conspicillata* and *Sylvia atricapilla*) that were not detected by the observational methods, but they were removed from the modelling analysis due to the small sample size. These results agree with previous studies that found DO to be more effective for detecting gregarious and large birds, such as doves and crows, and conspicuous species such as flycatchers^23,29,40^, while MN is more effective for detecting secretive and cryptic species^27,41,42^, such as warblers. Only small seed-eaters were detected with similar effectiveness by the three target methods. Importantly, MN showed the highest variability in the detection estimates and also led to wide differences in species detectability even within families and groups (Figs. 4, 5). This finding underlines the view that MN should not be used as a single method to study entire bird communities, mainly because of its low efficiency in detecting medium-sized and large birds.

In semi-arid environments, such as the Iberian southeast, water bodies exert a strong attractive pressure for terrestrial animals, and they offer an interesting chance to study biological communities. Small ponds in this semi-arid region are critical habitats for supporting biodiversity due the scarcity of free water resources available to wildlife^43^. The high proportion of bird species using our study ponds is a clear example of their contribution to biodiversity. The breeding bird community of the study area consists of around 120 species, excluding marine and wetland birds^36^. We recorded 57 breeding bird species using the small ponds, which represents 47.5% of the terrestrial breeding bird species in the whole study area. However, all the studied ponds were in mountainous areas dominated by Mediterranean forest, and no ponds from steppe lands or farmlands were included in the study design. Typical steppe birds, such as larks and sandgrouse, also probably use ponds located in open landscapes, so that an even higher richness of birds would be expected if all types of ponds found in the Iberian southeast were surveyed. Future studies that include ponds from open areas will improve our knowledge of the services offered by these critical habitats for the conservation of terrestrial birds. Whatever the case, we recommend the use of small ponds as a supplementary and additional tool in biological monitoring programmes in arid and semi-arid environments, since they increase the ability to collect more rigorous data. For example, the implementation of pond surveys in large monitoring programmes (such as breeding bird surveys or specific surveys focused on species of conservation concern) in semi-arid regions would complement data on species distribution and so contribute to conservation actions. The power of attraction of ponds for birds leads to a high proportion of species inhabiting their vicinity, because they can take advantage of one or more of the available resources (as water to drink or bathe in, and as a source of food), making them easier to detect. In this context, the use of multi-method modelling represents a useful approach to overcome problems regarding closure violations when directional movements of birds are occurring, and availability for detection may vary among surveys.

Our study points to the greater effectiveness of PV and DO compared to MN for detecting bird species. However, we recommend a rigorous evaluation of the most suitable sampling method during the design stage of any study because effectiveness will depend mainly on the study aims, the study area, the target species and the available resources. For example, DO need a high degree of skill, which must be equal for all observers if species identification is to be unequivocal^44^, demanding a high level of training in areas of great avian richness. However, DO is easier and faster to conduct than MN and generally demands less material, and both human and economic resources^45^, making it perhaps the most effective in terms of species detected per unit of effort^24,27,28^. Moreover, visual techniques are less invasive than MN and do not interfere with bird activity^29^.

On the other hand, the most novel method, PV, can increase the detection rates of given species in sites, such as ponds, where it is not possible to see the entire water surface so that some species may be overlooked, leading to incomplete data. In our case, the additional use of video cameras did not provide a significant improvement of detectability with what is possible with the simpler method of DO, although a weak trend was observed for some muscicapid species (*Luscinia megarhynchos*, *Saxicola torquata*, *Erithacus rubecula* and *Phoenicurus ochruros*) and thrush species (*Turdus merula* and *Turdus viscivorus*). For some of the above species, detection rates increased by more than 20% (*Saxicola torquata* and *Turdus viscivorus*) and even 50% (*Luscinia megarhynchos*) when video cameras were used as a complement to DO, but detection estimates showed wide confidence intervals which overlapped between DO and PV. The use of video cameras as a single method can reduce the sampling effort by covering several sampling sites simultaneously, but it is not always possible to cover the entire surface of the target habitat. Moreover, it should be noted that conventional cameras operate continuously and the lab time needed to review all recorded videos is considerable^32^. However, the method that involves most time and human resources is MN because at least two operators are required to reduce the time during which birds are handled. Nevertheless, MN provides an easy way to standardize sampling, decreasing surveyor bias, and to detect species that are often missed using other count methods, while enabling handling, thus providing individual information^24^. So, MN can provide very useful data for population management, such as breeding status, body condition or the sex-ratio of the target species^45^. For example, through MN conducted around some of the ponds studied, we obtained the first evidence of breeding by Hawfinch (*Coccothraustes coccothraustes*) and Common Redstart (*Phoenicurus phoenicurus*) in the study region. Accordingly, MN can be equally effective as DO to detect avian richness in habitats with high-density vegetation and low visibility conditions, such as reed beds. The additional and invaluable information obtained could well be regarded as compensating for the increased time and effort needed^45^. Evaluating the cost-effectiveness of different sampling methods, then, is recommended to match the available resources to the study aims. Our multiple-method modelling approach can be especially useful in multispecies conservation programmes, acting as a starting point to design accurate surveys that take into consideration incomplete detection.

## Methods

### Study area

This study was carried out in the province of Murcia, which is located in the southeast of the Iberian Peninsula. The study area covers 11,317 km^2^ and is one of the most arid zones in continental Europe^46^. Current annual precipitation is normally less than 350 mm in most of the Iberian southeast and this ecogeographical area is characterized by a strong water deficit during spring and summer. Despite its hydrological stress conditions, the study area comprises a varied set of environments that differ in climate, topography and vegetation. In general, the inland zones have a more continental climate, with colder winters and higher mean annual precipitation than the coastal zones. The Iberian southeast is mainly occupied by mosaics of agricultural and forest areas with different degrees of representativeness. During recent decades, land uses in this area have been increasingly devoted to intensive agricultural irrigation practices, which, together with the natural water scarcity, have led to the overexploitation of groundwater and surface water resources. This situation has dramatically decreased the free water available to wildlife^43^, especially in seasons of water deficit. Thus, the isolated small ponds still present in the study area, such as drinking troughs and artificial pools, play an essential role in supporting biodiversity^47–49^ and act as shelters for animal species linked to aquatic ecosystems^50^. Ponds provide several key services to terrestrial fauna such as surface water and food resources^51,52^. Therefore, these aquatic ecosystems have become useful model habitats in biodiversity studies due to their attraction for terrestrial animal species. In the present study, 19 small ponds extending across an inland-coastal gradient (Supplementary Fig. S3), and located in predominantly agro-forestry areas, were selected by convenience. The main criteria for selecting the water bodies studied were: (1) good access conditions for drinking terrestrial birds and their regular use by the avian community, and (2) the absence of pond features (surrounding habitat, vegetation cover, availability for birds, etc.) that would affect detectability. The selected sampling sites are mainly used for cattle and game-species watering.

### Sampling protocol

We recorded detection-non detection data from the 19 study ponds using direct observation (DO), video camera monitoring and mist netting (MN) captures. The ponds were surveyed three times with every sampling method, with some exceptions due to logistic or weather issues. Surveys were conducted in early-mid spring, late spring and early summer (from 28 March to 28 July 2017), covering the breeding season of birds in the study area. The sampling methods were successively applied at the study ponds, where DO and video monitoring were the first method applied to avoid possible behavioural changes in the birds caused by the more invasive MN method^44^. Direct observations were carried out in a portable hide deployed on the vegetation surrounding the ponds, where it was not expected to influence bird activity. The hide was at least 10 m from the pond and binoculars were used for species identification. All birds seen or heard in or around the study ponds (up to 10 m) were recorded. Conventional video cameras were used as a complement to DO, so that this combination of DO and video cameras was termed “direct observation plus video monitoring” (PV). Conventional video cameras (Panasonic Handycam, HC-V180, Panasonic Corporation, Osaka, Japan) were deployed in 10 sites, where an additional small pool (filling from the main pond) was not visible to the watchers. Cameras were positioned to cover the entire surface of the pools to ensure the birds were detected when drinking at any part of the edge of the water. Videos were later analysed in the laboratory by visualizing the entire video recordings.

Mist netting surveys were based on the use of three nets of 16 mm mesh (two measuring 2 × 12 m and the other 2 × 9 m) open in a 10 m radius round the ponds and deployed between the water and surrounding vegetation to decrease net visibility. Once captured each bird was ringed, measured (data not used in this study) and released. Mist netting was conducted in nine ponds where conditions were suitable to open the nets. Four mist-net days were missing due to adverse weather conditions. Mist net data were combined into a single detection history for each site.

Intervals between surveys at each site did not exceed 40 days and the survey order remained unchanged during the whole sampling period. In the study area, bird species of the coastal region show a slightly advanced breeding phenology due to warmer conditions. Thus, littoral ponds were the first sites to be surveyed in order to correct for this phenomenon. Each sampling lasted 3 h, beginning at sunrise and in good weather conditions^51^. The early morning period has been described as the time of greatest bird activity, after which species detectability steeply declines^25,28^. Moreover, surveys were conducted during rainless periods to avoid the strong decline in visiting rates of birds to ponds^32^. As mentioned, the three sampling methods were applied in similar conditions, and so it is assumed that they provide representative information about the bird community during the sampling period, while any difference in the results can be attributed to methodology^29^.

### Modelling framework

We generated method-specific detection histories for each breeding species recorded during the study period. Therefore, a maximum of nine detection events (three survey periods per three methods) were possible for each species. Species with less than five records or migratory non-breeding species were removed from the models in order to avoid bias and unreliable estimates related to small sample size^53,54^.

We used the multi-method occupancy modelling approach described by Nichols et al.^35^ to estimate species detectability. With this approach, method-specific detection probabilities can be calculated for two or more sampling methods^33,35^. The multi-method models also estimate two occupancy parameters that allow us to model the occupancy at two spatial scales, ψ and θ_*s*_. The large-scale occupancy parameter, ψ, describes the probability that the site is occupied by the species, while the occupancy parameters for the smaller scale, θ_*s*_, describe the probability that individuals of the target species are available for detection at the site, conditional on species presence^35^.

Six models were fitted to account for the variability derived from any interference of sampling methods and survey occasions in species detectability and small scale occupancy (Table 2). Because our study focuses on detectability, the large scale occupancy parameter, ψ, was always modelled as constant. A sin link was used in all cases.

**Table 2:** Multi-method occupancy models fitted to estimate detectability of bird species in ponds located in the province of Murcia.

Model	Description
ψ, θ, *p*	Null model
ψ, θ, *p*_*s*_	Constant θ, survey-dependent detectability
ψ, θ_*s*_, *p*	Survey-dependent θ_*s*_, constant detectability
ψ, θ, *p*_*m*_	Constant θ, method-dependent detectability
ψ, θ_*s*_, *p*_*m*_	Survey-dependent θ_*s*_, method-dependent detectability
ψ, θ_*s*_, *p*_*s*_	Survey-dependent θ_*s*_, survey-dependent detectability

In all cases the occupancy parameter ψ was modelled as constant. The small-scale occupancy parameters and the probabilities of detection were modelled as constant (θ, *p*), as specific of the survey (θ_*s*_, *p*_*s*_), and depending on the method (*p*_*m*_).

Differences in AICc (ΔAICc) between each model and the best one were used to rank models^55,56^ and establish the overall importance of each variable (sampling method and survey occasion) for explaining species detectability. Model-averaging of the six models allowed us to calculate the estimates of occupancy probability and detection probability for each species. All analyses were carried out with MARK (version 9.0)^57^ through the R interface package RMark (version 2.2.7)^58^.

The sampling protocol considered the analytical assumptions required to fit the multi-method occupancy model^35^, which allows the intervals between survey occasions to be open to changes in occupancy. The survey period lasted four months, from 28 March to 28 July, overlapping with the breeding season of all the terrestrial bird species of the study area. During this time, breeding species are settled in their breeding territories and large movements are not expected. Moreover, to meet the closure assumption, all migratory non-breeding species detected in the study area were removed from the modelling. We also assumed that occupancy was independent among study sites because the minimum distance between ponds was always greater than 1.5 km, which is a reasonable distance to consider sites as independent when the survey period covers the breeding season of birds.

Additionally, phylogenetic relatedness (family-level) and two ecological traits (body size and diet) of the recorded species were used to descriptively explore their influence on the species detectability, since both factors have previously reported to affect the detection process^15,54,59,60^. Body size and diet were used to allocate species to bird groups. Body mass was used as a measure of body size^29,54^, because it is a reasonable indicator of bird total size. Thus, bird species were grouped into three body size classes and four trophic classes, which can be found in Table 1. Life-history traits of the recorded species were obtained from Pearman et al.^61^.

Confidence intervals for parameter means were calculated using variances estimated by the delta method^62^, assuming that survey-specific estimates for each method were independent:

$$ \widehat{var}\left( {\overline{p}} \right) = \mathop \sum \limits_{i}^{n} \mathop \sum \limits_{j}^{s} \frac{1}{{\left( {n \cdot s} \right)^{2} }}\widehat{var}\left( {\overline{p}_{i,j} } \right), $$

where $\overline{p}_{i,j}$ is the estimated detection probability of species *i* and survey *j*, *n* is the number of parameters averaged for each survey and *s* is the number of survey occasions.

### Experiments on live vertebrates

All the field work activities were approved by the Dirección General de Medio Natural of the Autonomous Community of Murcia (reference number: AUF20170002), which regulates wildlife management in the study area. The ringing license was provided by the Spanish Ministry of Agriculture, Fisheries and Environment. This study was carried out in accordance with national and international guidelines for the care and use of animals.

## Supplementary Information

Supplementary Information 1.

Supplementary Information 2.

## Data Availability

The data supporting the results of this study are provided as Supplementary Data (a R workspace file: “mmRMark.RData”). This file contains a list-type object containing the occupancy data of the 36 bird species studied in RMark format^58^.

## References

[1] MacKenzie DI (2006). Occupancy Estimation and Modeling: Inferring Patterns and Dynamycs of Species Occurence.

[2] Kéry M, Royle JA (2016). Applied Hierarchical Modeling in Ecology.

[3] Lindenmayer DB (2012). Improving biodiversity monitoring. Austral Ecol..

[4] Einoder LD (2018). Occupancy and detectability modelling of vertebrates in northern Australia using multiple sampling methods. PLoS ONE.

[5] Boulinier T, Nichols JD, Sauer JR, Hines JE, Pollock KH (1998). Estimating species richness: The importance of heterogeneity in species detectability. Ecology.

[6] Tyre AJ, Tenhumberg B, Field SA, Niejalke D, Parris K (2003). Improving precision and reducing bias in biological surveys: Estimating false-negative error rates. Ecol. Appl..

[7] Kellner KF, Swihart RK (2014). Accounting for imperfect detection in ecology: A quantitative review. PLoS ONE.

[8] Iknayan KJ, Tingley MW, Furnas BJ, Beissinger SR (2014). Detecting diversity: Emerging methods to estimate species diversity. Trends Ecol. Evol..

[9] Kéry M, Schmidt B (2008). Imperfect detection and its consequences for monitoring for conservation. Community Ecol..

[10] Tingley MW, Beissinger SR (2013). Cryptic loss of montane avian richness and high community turnover over 100 years. Ecology.

[11] Leu M (2017). Effects of point-count duration on estimated detection probabilities and occupancy of breeding birds. J. F. Ornithol..

[12] Royle JA, Dorazio RM (2008). Hierarchical Modeling and Inference in Ecology. The Analysis of Data from Populations, Metapopulations and Communities.

[13] Guillera-Arroita G (2017). Modelling of species distributions, range dynamics and communities under imperfect detection: Advances, challenges and opportunities. Ecography.

[14] Kéry M, Royle JA, Plattner M, Dorazio RM (2009). Species richness and occupancy estimation in communities subject to temporary emigration. Ecology.

[15] Sólymos P, Matsuoka SM, Stralberg D, Barker NKS, Bayne EM (2018). Phylogeny and species traits predict bird detectability. Ecography.

[16] Jarzyna MA, Jetz W (2016). Detecting the multiple facets of biodiversity. Trends Ecol. Evol..

[17] Kéry M, Royle JA, Schmid H (2005). Modeling avian abundance from replicated counts. Ecol. Appl..

[18] Mackenzie DI, Royle JA (2005). Designing occupancy studies: General advice and allocating survey effort. J. Appl. Ecol..

[19] Jiménez-Franco MV (2018). Use of classical bird census transects as spatial replicates for hierarchical modeling of an avian community. Ecol. Evol..

[20] Clement MJ, Hines JE, Nichols JD, Pardieck KL, Ziolkowski DJ (2016). Estimating indices of range shifts in birds using dynamic models when detection is imperfect. Glob. Change Biol..

[21] Sliwinski M, Powell L, Koper N, Giovanni M, Schacht W (2016). Research design considerations to ensure detection of all species in an avian community. Methods Ecol. Evol..

[22] Rappole JH, Winker K, Powell GV (2012). Migratory bird habitat use in Southern Mexico: Mist nets versus point counts. J. F. Ornithol..

[23] Faaborg J, Arendt WJ, Dugger KM (2004). Bird population studies in Puerto Rico using mist nets: General patterns and comparisons with point counts. Stud. Avian Biol..

[24] Dunn EH, Ralph CJ (2004). Use of mist nets as a tool for bird population monitoring. Stud. Avian Biol..

[25] Lynch JF (1989). Distribution of overwintering Nearctic migrants in the Yucatan Peninsula, I: General patterns of occurrence. Condor.

[26] Wunderle JM, Waide RB (1993). Distribution of overwintering Nearctic migrants in the Bahamas and Greater Antilles. Condor.

[27] Gram WK, Faaborg J (1997). The distribution of neotropical migrant birds wintering in the El Cielo Biosphere Reserve, Tamaulipas, Mexico. Condor.

[28] Whitman AA, Hagan JM, Brokaw NVL (1997). A comparison of two bird survey techniques used in a subtropical forest. Condor.

[29] Arizaga, J., Deán, J. I., Vilches, A., Alonso, D. & Mendiburu, A. Monitoring communities of small birds: A comparison between mist-netting and counting. *Bird Study* **58**(3), 37–41 (2011).

[30] Darras K (2019). Autonomous sound recording outperforms human observation for sampling birds: A systematic map and user guide. Ecol. Appl..

[31] Smit B, Woodborne S, Wolf BO, McKechnie AE (2019). Differences in the use of surface water resources by desert birds are revealed using isotopic tracers. Auk.

[32] Lynn JC, Rosenstock SS, Chambers CL (2008). Avian use of desert wildlife water developments as determined by remote videography. West. N. Am. Nat..

[33] Fisher JT, Bradbury S (2014). A multi-method hierarchical modeling approach to quantifying bias in occupancy from noninvasive genetic tagging studies. J. Wildl. Manag..

[34] Fisher JT, Heim N, Code S, Paczkowski J (2016). Grizzly bear noninvasive genetic tagging surveys: Estimating the magnitude of missed detections. PLoS ONE.

[35] Nichols JD (2008). Multi-scale occupancy estimation and modelling using multiple detection methods. J. Appl. Ecol..

[36] Calvo JF (2017). Catálogo de las aves de la Región de Murcia (España). An. Biol..

[37] Galbraith JA, Jones DN, Beggs JR, Stanley MC, Parry K (2017). Urban bird feeders dominated by a few species and individuals. Front. Ecol. Evol..

[38] McCarthy MA (2012). The influence of abundance on detectability. Oikos.

[39] Lee ATK, Wright D, Barnard P (2017). Hot bird drinking patterns: Drivers of water visitation in a fynbos bird community. Afr. J. Ecol..

[40] Gregory, R. D., Gibbons, D. W. & Donald, P. F. Bird census and survey techniques. In *Bird Ecology and Conservation. A Handbook of Techniques* (eds. Sutherland, W. J., Newton, I. & Green, R. E.) 17–55 (Oxford Scholarship, Oxford, 2004).

[41] Derlindati EJ, Caziani SM (2005). Using canopy and understory mist nets and point counts to study bird assemblages in Chaco forests. Wilson Bull..

[42] Wang Y, Finch DM (2002). Consistency of mist netting and point counts in assessing landbird species richness and relative abundance during migration. Condor.

[43] Valera F (2011). History and adaptation stories of the vertebrate fauna of southern Spain semiarid habitats. J. Arid Environ..

[44] Rappole JH (2012). Migratory bird habitat use in Southern Mexico: Mist nets versus point counts. J. F. Ornithol..

[45] Poulin B, Lefebvre G, Pilard P (2000). Quantifying the breeding assemblage of reedbed passerines with mist-net and point-count surveys. J. F. Ornithol..

[46] Armas C, Miranda JD, Padilla FM, Pugnaire FI (2011). Special issue: The Iberian Southeast. J. Arid Environ..

[47] Lisón F, Calvo JF (2014). Bat activity over small ponds in dry Mediterranean forests: Implications for conservation. Acta Chiropterol..

[48] Sebastián-González E, Sánchez-Zapata JA, Botella F (2010). Agricultural ponds as alternative habitat for waterbirds: Spatial and temporal patterns of abundance and management strategies. Eur. J. Wildl. Res..

[49] Egea-Serrano A, Oliva-Paterna FJ, Torralva M (2006). Breeding habitat selection of *Salamandra salamandra* (Linnaeus, 1758) in the most arid zone of its European distribution range: Application to conservation management. Hydrobiologia.

[50] Egea-Serrano A, Oliva-Paterna FJ, Tejedo M, Torralva M (2006). Breeding habitat selection of an endangered species in an arid zone: The case of Alytes dickhilleni Arntzen & García-París, 1995. Acta Herpetol..

[51] Davies SR, Sayer CD, Greaves H, Siriwardena GM, Axmacher JC (2016). A new role for pond management in farmland bird conservation. Agric. Ecosyst. Environ..

[52] Oertli B (2018). Freshwater biodiversity conservation: The role of artificial ponds in the 21st century. Aquat. Conserv. Mar. Freshw. Ecosyst..

[53] MacKenzie DI (2002). Estimating site occupancy rates when detection probabilities are less than one. Ecology.

[54] Rich LN, Miller DAW, Robinson HS, McNutt JW, Kelly MJ (2016). Using camera trapping and hierarchical occupancy modelling to evaluate the spatial ecology of an African mammal community. J. Appl. Ecol..

[55] Burnham KP, Anderson DR (2002). Model Selection and Multimodel Inference: A Practical Information-Theoretic Approach.

[56] Martínez-Martí C, Jiménez-Franco MV, Royle JA, Palazón JA, Calvo JF (2016). Integrating occurrence and detectability patterns based on interview data: A case study for threatened mammals in Equatorial Guinea. Sci. Rep..

[57] White GC, Burnham KP (1999). Program MARK: survival estimation from populations of marked animals. Bird Study.

[58] Laake, J. L. *RMark: An R Interface for Analysis of Capture-Recapture Data with MARK*. AFSC Processed Report 2013–01, 25p. Alaska Fish. Sci. Cent., NOAA, Natl. Mar. Fish. Serv., 7600 Sand Point Way NE, Seattle WA 98115 (2013).

[59] Denis T (2017). Biological traits, rather than environment, shape detection curves of large vertebrates in neotropical rainforests. Ecol. Appl..

[60] Frishkoff LO, De Valpine P, M’Gonigle LK (2017). Phylogenetic occupancy models integrate imperfect detection and phylogenetic signal to analyze community structure. Ecology.

[61] Pearman PB (2014). Phylogenetic patterns of climatic, habitat and trophic niches in a European avian assemblage. Glob. Ecol. Biogeogr..

[62] Powell LA (2007). Approximating variance of demographic parameters using the delta method: A reference for avian biologists. Condor.

