# Supplementary material for: Comparing detectability patterns of bird species using multi-method occupancy modelling
Source: Sci Rep. 2021 Jan 28;11:2558. doi: 10.1038/s41598-021-81605-w (PMC7844255; doi:10.1038/s41598-021-81605-w)
Supplement: Supplementary file 1 — Supplementary Information 1. [file 41598_2021_81605_MOESM1_ESM.pdf]

Supplementary Information

Title: Comparing detectability patterns of bird species using multi-method occupancy modelling

Authors: José M. Zamora-Marín[1], Antonio Zamora-López[1], José F. Calvo[2], and Francisco J. Oliva-Paterna[1]

[1]Departamento de Zoología y Antropología Física, Facultad de Biología, Universidad de Murcia, Spain.

[2]Departamento de Ecología e Hidrología, Facultad de Biología, Universidad de Murcia, Spain.

Supplementary Figure S1. Bird species detectability at family level for each of the three surveys conducted in ponds in south-eastern Spain. Survey-specific, model-averaged estimates of detection probabilities (independent of the sampling method) grouped by species family. Vertical lines represent 95% confidence intervals. Only the five families with the highest number of recorded species are indicated, the remaining families being grouped as “other”. Families are indicated as follows: Emb, Emberizidae; Fri, Fringillidae; Mus, Muscicapidae; Oth, other families; Par, Paridae; and Syl, Sylviidae. Surveys 1, 2 and 3 correspond to visits conducted in early-mid spring, late spring and early summer, respectively. The figure was created in R (version 4.0.2, https://www.R-project.org/) and assembled with GIMP (version 2.10.14, https://www.gimp.org/).

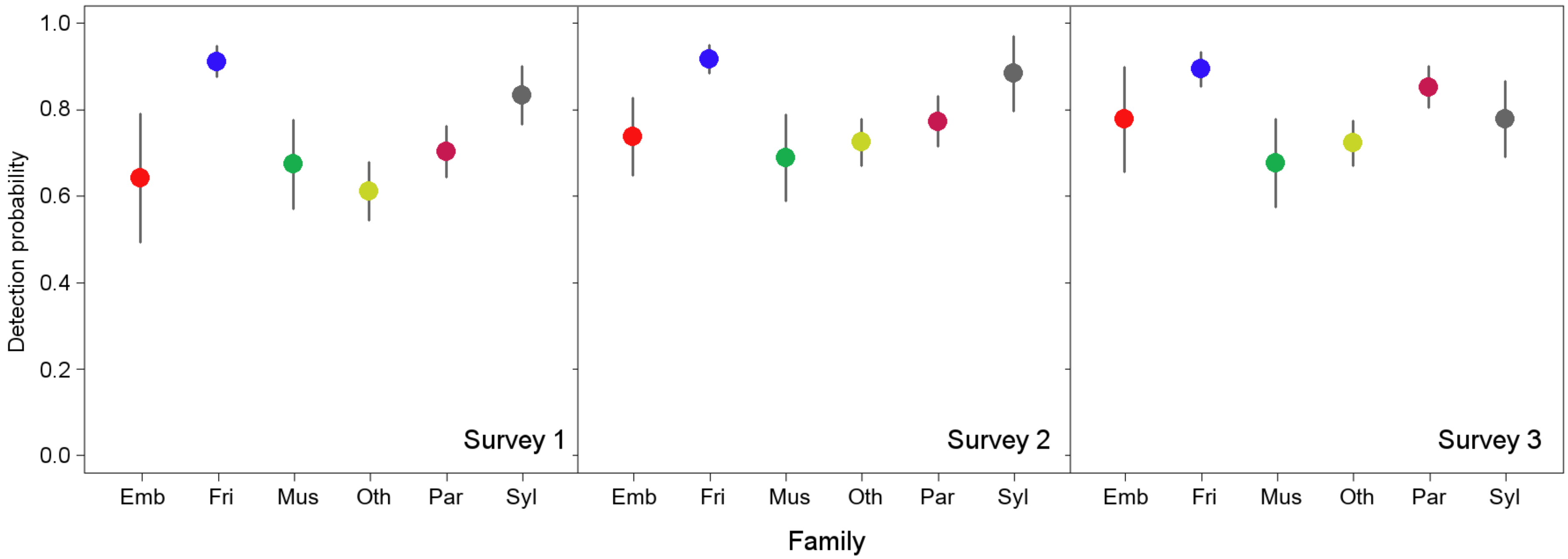

Supplementary Figure S2. Bird species detectability at group level for each of the three surveys conducted in ponds in south-eastern Spain. Survey-specific, model-averaged estimates of detection probabilities (independent of the sampling method) grouped by species group. Vertical lines represent 95% confidence intervals. Numbers refer to six different established groups based on body size and main diet type: 1, small insectivorous (< 30g); 2, medium-sized and large insectivorous (≥ 30g); 3, small insectivorous and frugivorous (< 30g); 4, small seed-eaters (< 30g): 5, medium-sized and large seed-eaters (≥ 30g); and 6, medium-sized and large generalists (≥ 30g). Surveys 1, 2 and 3 correspond to visits conducted in early-mid spring, late spring and early summer, respectively. The figure was created in R (version 4.0.2, https://www.R-project.org/) and assembled with GIMP (version 2.10.14, https://www.gimp.org/).

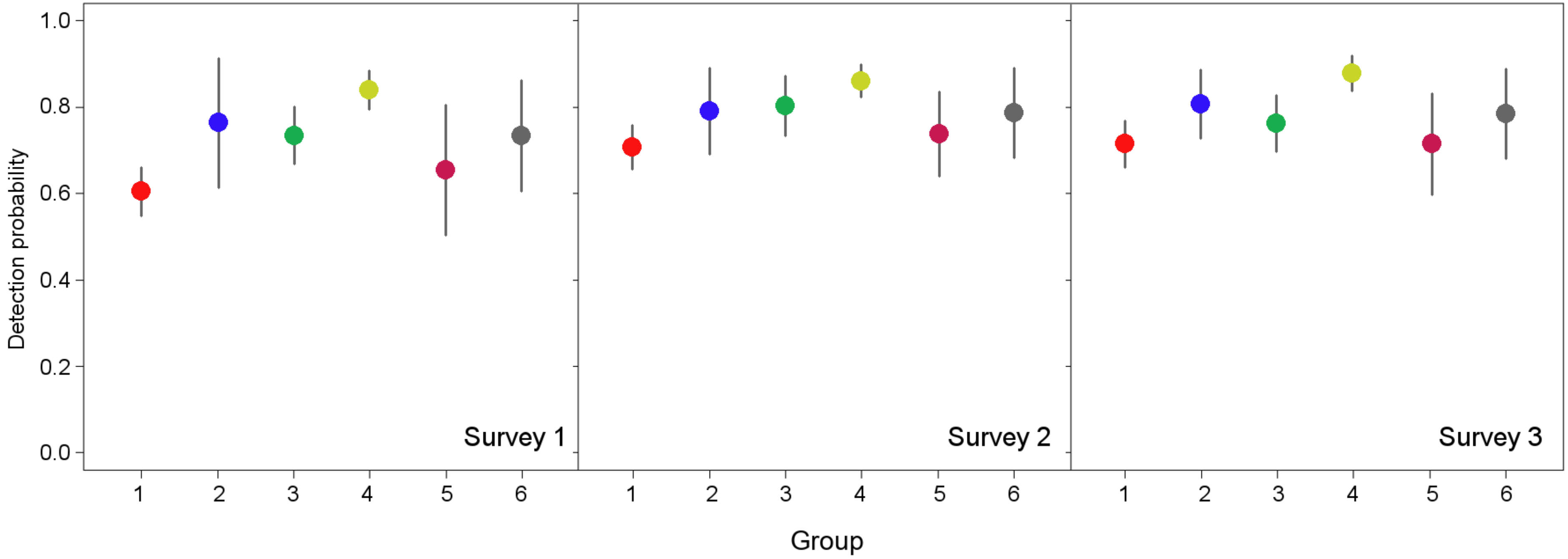

Supplementary Figure S3. Location map of the study ponds. Coordinates are indicated as UTM 30S (metres). Elevation data and outline maps were obtained from public national data sources (https://www.ign.es/web/cbg-area-cartografia). The maps were drawn in R (version 4.0.2, https://www.R-project.org/) by using the package *raster* (https://cran.r-project.org/web/packages/raster/), and the figure was assembled with GIMP (version 2.10.14, https://www.gimp.org/).

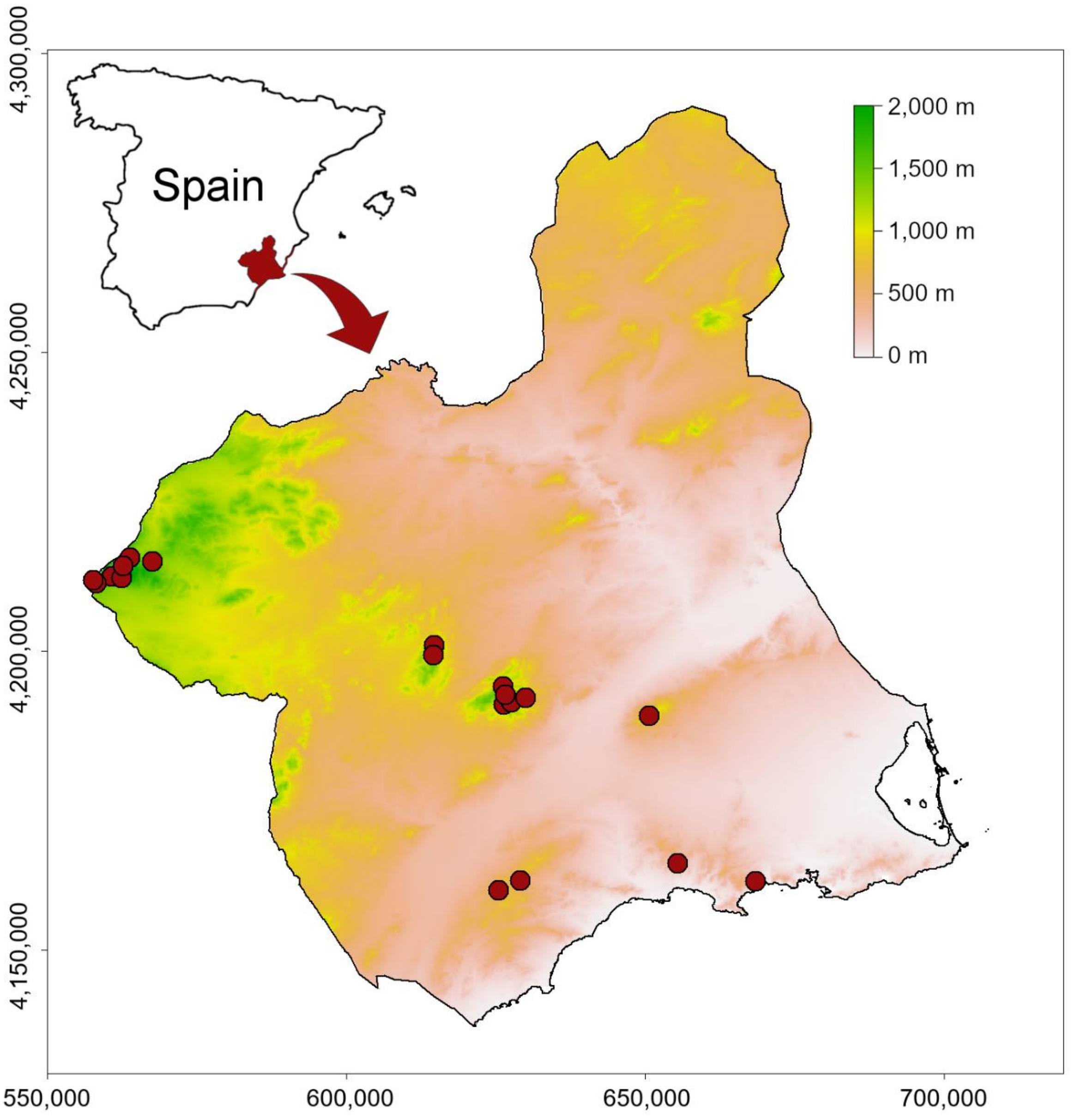

Supplementary Table S1. Occupancy estimates and best models for 36 bird species recorded in pond surveys in south-eastern Spain. Model-averaged estimates of the occupancy parameters (ψ and θ_s) are given for each species, with their associated standard errors in parentheses. In all cases the occupancy parameter ψ was modelled as constant. The small-scale occupancy parameters and the probabilities of detection were modelled as constant (θ, p), as specific of the survey (θ_s, p_s), and depending on the method (p_m). Best models were selected according to the lowest Akaike Information Criterion value. Species are ranked by phylogenetic order.

**Species**	**ψ**	**θ_1**	**θ_2**	**θ_3**	**Best model**
Columba palumbus	0.770 (0.133)	0.565 (0.108)	0.567 (0.109)	0.564 (0.108)	ψ, θ, p_m
Streptopelia turtur	0.236 (0.106)	0.424 (14.159)	0.785 (0.269)	0.876 (0.216)	ψ, θ_s, p_s
Turdus viscivorus	0.445 (0.141)	0.639 (0.168)	0.634 (0.169)	0.649 (0.176)	ψ, θ, p_m
Turdus merula	1.000 (0.000)	0.829 (0.100)	0.855 (0.080)	0.884 (0.083)	ψ, θ, p_m
Luscinia megarhynchos	0.336 (0.137)	0.705 (0.226)	0.724 (0.222)	0.708 (0.226)	ψ, θ, p_m
Erithacus rubecula	0.455 (0.123)	0.842 (0.108)	0.855 (0.105)	0.842 (0.108)	ψ, θ, p_m
Phoenicurus ochruros	0.315 (0.171)	0.437 (0.220)	0.419 (0.212)	0.426 (0.210)	ψ, θ, p
Saxicola torquata	0.278 (0.107)	0.761 (0.316)	0.926 (0.197)	0.926 (0.197)	ψ, θ, p_m
Muscicapa striata	0.184 (0.102)	0.604 (0.266)	0.630 (0.274)	0.638 (0.264)	ψ, θ, p_m
Hippolais polyglotta	0.484 (0.117)	0.971 (0.121)	0.997 (0.024)	0.997 (0.024)	ψ, θ, p_s
Phylloscopus collybita	1.000 (0.000)	0.158 (0.089)	0.148 (0.082)	0.123 (0.087)	ψ, θ, p
Phylloscopus bonelli	0.463 (0.123)	0.835 (0.265)	0.906 (0.116)	0.931 (0.089)	ψ, θ, p_s
Sylvia hortensis	0.241 (0.109)	0.835 (0.181)	0.816 (0.195)	0.818 (0.195)	ψ, θ, p_s
Sylvia undata	1.000 (0.000)	0.085 (0.042)	0.087 (0.044)	0.085 (0.042)	ψ, θ, p
Sylvia cantillans	0.720 (0.134)	0.594 (0.118)	0.633 (0.137)	0.597 (0.116)	ψ, θ, p
Sylvia melanocephala	0.396 (0.121)	0.695 (0.133)	0.676 (0.142)	0.695 (0.133)	ψ, θ, p
Periparus ater	0.778 (0.121)	0.749 (0.107)	0.737 (0.104)	0.735 (0.107)	ψ, θ, p
Lophophanes cristatus	0.805 (0.096)	0.879 (0.072)	0.877 (0.072)	0.877 (0.072)	ψ, θ, p_s
Parus major	0.921 (0.076)	0.886 (0.097)	0.891 (0.092)	0.903 (0.088)	ψ, θ, p_s

**Species**	**ψ**	**θ₁**	**θ₂**	**θ₃**	**Best model**
Cyanistes caeruleus	0.623 (0.124)	0.751 (0.132)	0.781 (0.114)	0.814 (0.123)	ψ, θ, *p*
Aegithalos caudatus	0.575 (0.150)	0.667 (0.165)	0.671 (0.169)	0.665 (0.164)	ψ, θ, *p*
Sitta europaea	0.142 (0.108)	0.503 (0.319)	0.446 (0.338)	0.575 (0.334)	ψ, θ, *p*
Certhia brachydactyla	0.544 (0.306)	0.496 (0.355)	0.456 (0.330)	0.497 (0.360)	ψ, θ, *p*
Lanius senator	0.177 (0.097)	0.496 (0.375)	0.605 (0.315)	0.738 (0.291)	ψ, θ, *p*
Garrulus glandarius	0.846 (0.107)	0.683 (0.101)	0.680 (0.102)	0.681 (0.101)	ψ, θ, p_m
Pica pica	0.279 (0.144)	0.393 (0.221)	0.409 (0.224)	0.544 (0.291)	ψ, θ, p_m
Petronia petronia	0.710 (0.319)	0.312 (0.166)	0.314 (0.167)	0.312 (0.166)	ψ, θ, *p*
Fringilla coelebs	0.954 (0.053)	0.915 (0.063)	0.918 (0.063)	0.920 (0.061)	ψ, θ, *p*
Serinus serinus	0.962 (0.054)	0.877 (0.059)	0.879 (0.061)	0.873 (0.063)	ψ, θ, *p*
Carduelis chloris	0.351 (0.121)	0.666 (0.140)	0.669 (0.137)	0.675 (0.138)	ψ, θ, *p*
Carduelis carduelis	0.775 (0.121)	0.659 (0.135)	0.704 (0.116)	0.779 (0.156)	ψ, θ, *p*
Linaria cannabina	0.825 (0.109)	0.749 (0.091)	0.751 (0.092)	0.746 (0.094)	ψ, θ, p_s
Loxia curvirostra	0.875 (0.104)	0.654 (0.089)	0.652 (0.089)	0.653 (0.089)	ψ, θ, p_m
Emberiza calandra	1.000 (0.000)	0.117 (294.256)	0.215 (0.133)	0.141 (0.117)	ψ, θ, *p*
Emberiza cia	0.882 (0.090)	0.778 (0.120)	0.792 (0.109)	0.793 (0.105)	ψ, θ, p_s
Emberiza cirlus	0.782 (0.119)	0.618 (0.165)	0.897 (0.140)	0.749 (0.130)	ψ, $θ_s$, *p*

Supplementary Table S2. Model comparisons to identify covariates (survey and/or method) influencing detectability of 36 bird species in pond surveys in south-eastern Spain. The total number of estimable parameters (*K*), the Akaike Information Criterion (AICc), the relative differences in AICc (ΔAICc), the Akaike weights (*w*) and model deviance are given for each species. In all cases the occupancy parameter ψ was modelled as constant. The small-scale occupancy parameters and the probabilities of detection (θ and *p*, respectively) were modelled as constant, as specific of the survey (θ_s, p_s), and depending on the method (p_m). Species are ranked by phylogenetic order.

Species	Model	*K*	AICc	ΔAICc	*w*	Deviance
Columba palumbus	ψ, θ, p_m	5	79.09	0.00	0.9850	64.48
	ψ, θ_s, p_m	7	87.51	8.42	0.0150	63.33
	ψ, θ, p	3	109.93	30.83	0.0000	102.33
	ψ, θ_s, p	5	115.49	36.40	0.0000	100.88
	ψ, θ, p_s	5	116.26	37.16	0.0000	101.64
	ψ, θ_s, p_s	7	124.63	45.54	0.0000	100.45
Streptopelia turtur	ψ, θ_s, p_m	7	47.45	0.00	0.2420	23.27
	ψ, θ, p_s	5	47.46	0.00	0.2420	32.84
	ψ, θ, p_m	5	47.54	0.09	0.2320	32.92
	ψ, θ_s, p	5	47.76	0.31	0.2080	33.15
	ψ, θ, p	3	49.84	2.39	0.0730	42.24
	ψ, θ_s, p_s	7	57.02	9.57	0.0020	32.84
Turdus viscivorus	ψ, θ, p_m	5	84.56	0.00	0.5461	69.95
	ψ, θ, p	3	85.19	0.63	0.3993	77.59
	ψ, θ_s, p	5	90.69	6.13	0.0254	76.08
	ψ, θ, p_s	5	91.49	6.93	0.0171	76.87
	ψ, θ_s, p_m	7	92.22	7.66	0.0118	68.04
	ψ, θ_s, p_s	7	99.98	15.42	0.0002	75.80
Turdus merula	ψ, θ, p_m	5	125.31	0.00	0.5946	110.69
	ψ, θ, p	3	127.37	2.06	0.2121	119.77
	ψ, θ_s, p_m	7	129.03	3.72	0.0926	104.84
	ψ, θ_s, p	5	129.05	3.75	0.0913	114.44
	ψ, θ, p_s	5	133.77	8.47	0.0086	119.16
	ψ, θ_s, p_s	7	138.60	13.30	0.0008	114.42
Luscinia megarhynchos	ψ, θ, p_m	5	58.27	0.00	0.8144	43.66
	ψ, θ, p	3	62.24	3.96	0.1122	54.64
	ψ, θ, p_s	5	64.56	6.29	0.0351	49.94
	ψ, θ_s, p_m	7	65.06	6.79	0.0273	40.88
	ψ, θ_s, p	5	66.96	8.69	0.0106	52.34
	ψ, θ_s, p_s	7	74.08	15.80	0.0003	49.89
Erithacus rubecula	ψ, θ, p_m	5	94.95	0.00	0.5245	80.33
	ψ, θ, p	3	95.97	1.03	0.3141	88.37
	ψ, θ, p_s	5	98.13	3.19	0.1064	83.52
	ψ, θ_s, p	5	100.36	5.41	0.0350	85.74
	ψ, θ_s, p_m	7	101.76	6.82	0.0173	77.58
	ψ, θ_s, p_s	7	105.54	10.59	0.0026	81.35

Species	Model	K	AICc	ΔAICc	w	Deviance
Phoenicurus ochruros	ψ, θ, p	3	57.39	0.00	0.7294	49.79
	ψ, θ, p_m	5	60.45	3.06	0.1581	45.84
	ψ, θ, p_s	5	62.17	4.77	0.0670	47.55
	ψ, θ_s, p	5	63.14	5.74	0.0413	48.52
	ψ, θ_s, p_m	7	68.63	11.24	0.0026	44.45
	ψ, θ_s, p_s	7	69.71	12.32	0.0015	45.53
Saxicola torquata	ψ, θ, p_m	5	63.33	0.00	0.4579	48.72
	ψ, θ, p	3	64.62	1.28	0.2410	57.02
	ψ, θ_s, p	5	65.00	1.67	0.1987	50.39
	ψ, θ_s, p_m	7	67.02	3.69	0.0724	42.84
	ψ, θ, p_s	5	69.08	6.15	0.0212	54.86
	ψ, θ_s, p_s	7	71.23	7.89	0.0088	47.04
Muscicapa striata	ψ, θ, p_m	5	40.95	0.00	0.5491	26.33
	ψ, θ, p	3	41.73	0.79	0.3703	34.13
	ψ, θ_s, p	5	46.34	5.39	0.0370	31.73
	ψ, θ, p_s	5	46.93	5.98	0.0276	32.31
	ψ, θ_s, p_m	7	48.06	7.12	0.0156	23.88
	ψ, θ_s, p_s	7	55.31	14.36	0.0004	31.13
Hippolais polyglotta	ψ, θ, p_s	5	59.89	0.00	0.9253	45.27
	ψ, θ_s, p	5	66.32	6.44	0.0370	51.71
	ψ, θ, p	3	68.25	8.37	0.0141	60.65
	ψ, θ_s, p_s	7	68.46	8.57	0.0128	44.27
	ψ, θ_s, p_m	7	69.78	9.89	0.0066	45.60
	ψ, θ, p_m	5	70.65	10.76	0.0043	56.03
Phylloscopus collybita	ψ, θ, p	3	50.26	0.00	0.6078	42.66
	ψ, θ, p_s	5	52.55	2.29	0.1933	37.94
	ψ, θ_s, p	5	53.00	2.74	0.1542	38.39
	ψ, θ, p_m	5	55.75	5.49	0.0391	41.13
	ψ, θ_s, p_s	7	60.99	10.73	0.0028	36.81
Phylloscopus bonelli	ψ, θ, p_s	5	76.28	0.00	0.8681	61.66
	ψ, θ_s, p	5	80.43	4.15	0.1088	65.82
	ψ, θ_s, p_s	7	83.82	7.54	0.0201	59.63
	ψ, θ, p	3	88.39	12.11	0.0020	80.79
	ψ, θ_s, p_m	7	89.80	13.52	0.0010	65.62
	ψ, θ, p_m	5	95.25	18.97	0.0001	80.64
Sylvia hortensis	ψ, θ, p_s	5	62.26	0.00	0.5222	47.64
	ψ, θ, p	3	62.79	0.53	0.3998	55.19
	ψ, θ_s, p	5	67.54	5.28	0.0372	52.92
	ψ, θ, p_m	5	68.29	6.04	0.0255	53.68
	ψ, θ_s, p_s	7	69.43	7.17	0.0145	45.25
	ψ, θ_s, p_m	7	75.47	13.21	0.0007	51.29
Sylvia undata	ψ, θ, p	3	41.24	0.00	0.7677	33.64
	ψ, θ, p_m	5	45.10	3.86	0.1115	30.48
	ψ, θ, p_s	5	45.56	4.33	0.0882	30.95
	ψ, θ_s, p	5	47.69	6.45	0.0305	33.08
	ψ, θ_s, p_m	7	54.10	12.86	0.0012	29.92
	ψ, θ_s, p_s	7	54.68	13.44	0.0009	30.50

Species	Model	K	AICc	ΔAICc	w	Deviance
Sylvia cantillans	ψ, θ, p	3	91.94	0.00	0.6135	84.34
	ψ, θ, p_s	5	94.57	2.63	0.1647	79.95
	ψ, θ, p_m	5	95.38	3.44	0.1098	80.76
	ψ, θ_s, p	5	95.62	3.69	0.0971	81.01
	ψ, θ_s, p_s	7	100.13	8.20	0.0102	75.95
	ψ, θ_s, p_m	7	101.67	9.73	0.0047	77.49
Sylvia melanocephala	ψ, θ, p	3	61.10	0.00	0.7748	53.50
	ψ, θ, p_m	5	65.00	3.90	0.1104	50.38
	ψ, θ_s, p	5	66.34	5.24	0.0564	51.73
	ψ, θ, p_s	5	66.40	5.29	0.0549	51.78
	ψ, θ_s, p_m	7	72.70	11.60	0.0023	48.52
	ψ, θ_s, p_s	7	74.02	12.92	0.0012	49.84
Periparus ater	ψ, θ, p	3	115.76	0.00	0.8215	108.16
	ψ, θ, p_m	5	120.57	4.80	0.0744	105.95
	ψ, θ_s, p	5	121.13	5.37	0.0560	106.52
	ψ, θ, p_s	5	121.54	5.78	0.0457	106.93
	ψ, θ_s, p_m	7	128.63	12.87	0.0013	104.45
	ψ, θ_s, p_s	7	128.95	13.19	0.0011	104.77
Lophophanes cristatus	ψ, θ, p_s	5	114.70	0.00	0.9152	100.08
	ψ, θ, p	3	119.96	5.26	0.0660	112.36
	ψ, θ_s, p_s	7	123.38	8.68	0.0119	99.20
	ψ, θ, p_m	5	125.27	10.57	0.0046	110.66
	ψ, θ_s, p	5	126.76	12.06	0.0022	112.14
	ψ, θ_s, p_m	7	134.68	19.98	0.0000	110.49
Parus major	ψ, θ, p_s	5	120.83	0.00	0.6269	106.21
	ψ, θ, p	3	122.38	1.56	0.2880	114.78
	ψ, θ_s, p	5	125.61	4.79	0.0572	111.00
	ψ, θ, p_m	5	128.00	7.18	0.0173	113.39
	ψ, θ_s, p_s	7	129.22	8.39	0.0094	105.03
	ψ, θ_s, p_m	7	133.62	12.79	0.0010	109.43
Cyanistes caeruleus	ψ, θ, p	3	107.16	0.00	0.6114	99.56
	ψ, θ, p_s	5	109.32	2.16	0.2072	94.71
	ψ, θ_s, p	5	110.09	2.93	0.1414	95.47
	ψ, θ, p_m	5	113.75	6.59	0.0226	99.14
	ψ, θ_s, p_s	7	114.46	7.30	0.0159	90.28
	ψ, θ_s, p_m	7	119.35	12.19	0.0014	95.17
Aegithalos caudatus	ψ, θ, p	3	98.73	0.00	0.8017	91.13
	ψ, θ, p_s	5	103.22	4.49	0.0850	88.60
	ψ, θ, p_m	5	103.28	4.55	0.0825	88.66
	ψ, θ_s, p	5	105.40	6.67	0.0286	90.78
	ψ, θ_s, p_s	7	111.26	12.53	0.0015	87.08
	ψ, θ_s, p_m	7	112.66	13.93	0.0008	88.47
Sitta europaea	ψ, θ, p	3	34.34	0.00	0.6148	26.74
	ψ, θ, p_m	5	37.31	2.97	0.1394	22.69
	ψ, θ_s, p	5	37.62	3.28	0.1191	23.01
	ψ, θ, p_s	5	37.66	3.32	0.1169	23.04
	ψ, θ_s, p_m	7	43.18	8.84	0.0074	19.00
	ψ, θ_s, p_s	7	45.42	11.08	0.0024	21.24

Species	Model	*K*	AICc	ΔAICc	*w*	Deviance
Certhia brachydactyla	ψ, θ, p	3	63.17	0.00	0.8357	55.57
	ψ, θ_s, p	5	68.09	4.92	0.0714	53.48
	ψ, θ, p_m	5	68.60	5.43	0.0553	53.99
	ψ, θ, p_s	5	69.76	6.59	0.0309	55.15
	ψ, θ_s, p_s	7	73.31	10.14	0.0052	49.13
	ψ, θ_s, p_m	7	75.92	12.75	0.0014	51.74
Lanius senator	ψ, θ, p	3	31.27	0.00	0.6170	23.67
	ψ, θ_s, p	5	33.19	1.92	0.2360	18.58
	ψ, θ, p_s	5	34.47	3.20	0.1250	19.85
Garrulus glandarius	ψ, θ, p_m	5	111.48	0.00	0.9780	96.86
	ψ, θ_s, p_m	7	119.77	8.29	0.0155	95.59
	ψ, θ, p	3	121.69	10.22	0.0059	114.09
	ψ, θ_s, p	5	127.41	15.93	0.0003	112.80
	ψ, θ, p_s	5	127.99	16.51	0.0003	113.37
	ψ, θ_s, p_s	7	135.61	24.13	0.0000	111.43
Pica pica	ψ, θ, p_m	5	47.49	0.00	0.5994	32.88
	ψ, θ, p	3	49.75	2.26	0.1937	42.15
	ψ, θ_s, p_m	7	51.11	3.61	0.0984	26.93
	ψ, θ_s, p	5	51.16	3.66	0.0960	36.54
	ψ, θ, p_s	5	55.63	8.14	0.0102	41.02
	ψ, θ_s, p_s	7	58.73	11.23	0.0022	34.55
Petronia petronia	ψ, θ, p	3	68.73	0.00	0.5977	61.13
	ψ, θ, p_s	5	69.89	1.17	0.3333	55.28
	ψ, θ, p_m	5	73.91	5.19	0.0447	59.30
	ψ, θ_s, p	5	75.45	6.72	0.0208	60.83
	ψ, θ_s, p_s	7	79.17	10.45	0.0032	54.99
	ψ, θ_s, p_m	7	83.20	14.48	0.0004	59.02
Fringilla coelebs	ψ, θ, p	3	79.32	0.00	0.6425	71.72
	ψ, θ, p_s	5	81.70	2.38	0.1953	67.08
	ψ, θ, p_m	5	82.62	3.30	0.1234	68.00
	ψ, θ_s, p	5	85.15	5.83	0.0348	70.53
	ψ, θ_s, p_m	7	90.49	11.17	0.0024	66.31
	ψ, θ_s, p_s	7	91.22	11.90	0.0017	67.03
Serinus serinus	ψ, θ, p	3	68.21	0.00	0.7217	60.61
	ψ, θ, p_m	5	71.10	2.89	0.1701	56.49
	ψ, θ, p_s	5	73.02	4.81	0.0651	58.41
	ψ, θ_s, p	5	74.01	5.80	0.0398	59.39
	ψ, θ_s, p_m	7	79.52	11.31	0.0025	55.34
	ψ, θ_s, p_s	7	81.92	13.71	0.0008	57.74
Carduelis chloris	ψ, θ, p	3	58.75	0.00	0.7839	51.15
	ψ, θ, p_s	5	62.37	3.63	0.1279	47.76
	ψ, θ, p_m	5	64.21	5.46	0.0511	49.59
	ψ, θ_s, p	5	64.96	6.22	0.0350	50.35
	ψ, θ_s, p_s	7	71.38	12.64	0.0014	47.20
	ψ, θ_s, p_m	7	73.04	14.29	0.0006	48.86

**Species**	**Model**	***K***	**AICc**	**ΔAICc**	***w***	**Deviance**
Carduelis carduelis	ψ, θ, p	3	110.65	0.00	0.4470	103.05
	ψ, θ_s, p	5	111.99	1.34	0.2290	97.37
	ψ, θ, p_s	5	112.25	1.60	0.2010	97.64
	ψ, θ, p_m	5	113.80	3.15	0.0930	99.19
	ψ, θ_s, p_s	7	117.06	6.41	0.0180	92.88
	ψ, θ_s, p_m	7	117.97	7.31	0.0120	93.78
Linaria cannabina	ψ, θ, p_s	5	100.17	0.00	0.6248	85.55
	ψ, θ, p_m	5	102.31	2.14	0.2138	87.70
	ψ, θ, p	3	103.16	2.99	0.1398	95.56
	ψ, θ_s, p	5	108.33	8.17	0.0105	93.72
	ψ, θ_s, p_s	7	109.29	9.12	0.0065	85.10
	ψ, θ_s, p_m	7	110.03	9.86	0.0045	85.85
Loxia curvirostra	ψ, θ, p_m	5	106.54	0.00	0.9493	91.93
	ψ, θ, p	3	113.06	6.51	0.0366	105.46
	ψ, θ_s, p_m	7	115.49	8.95	0.0108	91.31
	ψ, θ_s, p	5	119.12	12.58	0.0018	104.50
	ψ, θ, p_s	5	119.44	12.90	0.0015	104.83
	ψ, θ_s, p_s	7	127.82	21.27	0.0000	103.64
Emberiza calandra	ψ, θ, p	3	48.30	0.00	0.3974	40.70
	ψ, θ_s, p	5	48.60	0.30	0.3424	33.98
	ψ, θ, p_s	5	49.32	1.02	0.2381	34.71
	ψ, θ, p_m	5	54.88	6.58	0.0148	40.26
	ψ, θ_s, p_m	7	57.67	9.37	0.0037	33.48
	ψ, θ_s, p_s	7	57.72	9.42	0.0036	33.53
Emberiza cia	ψ, θ, p_s	5	110.89	0.00	0.8673	96.28
	ψ, θ, p	3	115.72	4.83	0.0775	108.12
	ψ, θ_s, p_s	7	117.98	7.08	0.0251	93.79
	ψ, θ_s, p	5	118.49	7.59	0.0195	103.87
	ψ, θ, p_m	5	119.85	8.96	0.0098	105.23
	ψ, θ_s, p_m	7	125.04	14.14	0.0007	100.86
Emberiza cirlus	ψ, θ_s, p	5	118.83	0.00	0.5819	104.22
	ψ, θ, p	3	119.77	0.94	0.3638	112.17
	ψ, θ, p_s	5	125.56	6.73	0.0201	110.94
	ψ, θ, p_m	5	125.75	6.92	0.0183	111.14
	ψ, θ_s, p_m	7	127.28	8.45	0.0085	103.10
	ψ, θ_s, p_s	7	127.55	8.72	0.0074	103.37

Supplementary Table S3. Detection probabilities for 36 bird species in pond surveys in south-eastern Spain. Survey-specific, model averaged detection estimates from mist netting (MN), direct observation (DO) and direct observation plus video monitoring (PV) methods are given for each species, with their associated standard errors in parentheses. Species are ranked by phylogenetic order.

	Mist netting (MN)			Direct observation (DO)			Direct observation plus video monitoring (PV)		
**Species**	**Survey 1**	**Survey 2**	**Survey 3**	**Survey 1**	**Survey 2**	**Survey 3**	**Survey 1**	**Survey 2**	**Survey 3**
Columba palumbus	0.000 (0.000)	0.000 (0.000)	0.000 (0.000)	1.000 (0.000)	1.000 (0.000)	1.000 (0.000)	1.000 (0.000)	1.000 (0.000)	1.000 (0.000)
Streptopelia turtur	0.221 (0.361)	0.392 (0.391)	0.428 (0.420)	0.696 (0.412)	0.867 (0.173)	0.903 (0.137)	0.696 (0.412)	0.867 (0.173)	0.903 (0.137)
Turdus viscivorus	0.486 (0.183)	0.486 (0.184)	0.489 (0.184)	0.686 (0.148)	0.686 (0.150)	0.689 (0.146)	0.832 (0.205)	0.832 (0.206)	0.835 (0.201)
Turdus merula	0.598 (0.141)	0.599 (0.141)	0.600 (0.141)	0.779 (0.074)	0.779 (0.072)	0.780 (0.072)	0.879 (0.108)	0.880 (0.106)	0.880 (0.106)
Luscinia megarhynchos	0.066 (0.170)	0.084 (0.208)	0.071 (0.177)	0.441 (0.180)	0.459 (0.183)	0.446 (0.177)	0.907 (0.227)	0.926 (0.188)	0.912 (0.214)
Erithacus rubecula	0.639 (0.162)	0.653 (0.166)	0.610 (0.159)	0.777 (0.111)	0.791 (0.099)	0.748 (0.138)	0.886 (0.147)	0.899 (0.127)	0.857 (0.186)
Phoenicurus ochruros	0.658 (0.172)	0.687 (0.185)	0.670 (0.176)	0.704 (0.155)	0.733 (0.161)	0.717 (0.155)	0.741 (0.173)	0.770 (0.172)	0.754 (0.170)
Saxicola torquata	0.603 (0.268)	0.606 (0.265)	0.613 (0.270)	0.787 (0.181)	0.790 (0.175)	0.797 (0.174)	0.911 (0.124)	0.914 (0.112)	0.922 (0.101)
Muscicapa striata	0.279 (0.352)	0.290 (0.356)	0.294 (0.360)	0.844 (0.233)	0.855 (0.211)	0.859 (0.208)	0.844 (0.233)	0.855 (0.211)	0.859 (0.208)
Hippolais polyglotta	0.514 (0.168)	0.994 (0.035)	0.994 (0.035)	0.517 (0.173)	0.997 (0.018)	0.997 (0.018)	0.517 (0.173)	0.997 (0.018)	0.997 (0.018)
Phylloscopus collybita	0.546 (0.235)	0.601 (0.234)	0.461 (0.300)	0.541 (0.232)	0.596 (0.232)	0.456 (0.296)	0.562 (0.245)	0.618 (0.240)	0.478 (0.312)
Phylloscopus bonelli	0.145 (0.240)	0.888 (0.108)	0.817 (0.093)	0.145 (0.240)	0.888 (0.108)	0.817 (0.093)	0.144 (0.240)	0.887 (0.108)	0.817 (0.093)
Sylvia hortensis	0.568 (0.155)	0.790 (0.248)	0.381 (0.215)	0.570 (0.154)	0.791 (0.245)	0.382 (0.216)	0.577 (0.157)	0.798 (0.241)	0.389 (0.224)
Sylvia undata	0.836 (0.208)	0.836 (0.208)	0.797 (0.236)	0.893 (0.127)	0.893 (0.127)	0.854 (0.181)	0.893 (0.127)	0.893 (0.127)	0.854 (0.181)
Sylvia cantillans	0.922 (0.075)	0.894 (0.079)	0.922 (0.075)	0.935 (0.054)	0.907 (0.065)	0.935 (0.054)	0.944 (0.052)	0.916 (0.067)	0.944 (0.052)
Sylvia melanocephala	0.933 (0.091)	0.939 (0.089)	0.939 (0.089)	0.956 (0.052)	0.962 (0.045)	0.962 (0.045)	0.956 (0.052)	0.962 (0.045)	0.962 (0.045)
Periparus ater	0.788 (0.072)	0.785 (0.076)	0.793 (0.076)	0.790 (0.069)	0.786 (0.073)	0.795 (0.073)	0.803 (0.074)	0.799 (0.079)	0.807 (0.077)
Lophophanes cristatus	0.518 (0.123)	0.873 (0.082)	0.921 (0.077)	0.518 (0.123)	0.873 (0.082)	0.921 (0.077)	0.518 (0.125)	0.874 (0.082)	0.922 (0.076)

	Mist netting (MN)			Direct observation (DO)			Direct observation plus video monitoring (PV)		
**Species**	**Survey 1**	**Survey 2**	**Survey 3**	**Survey 1**	**Survey 2**	**Survey 3**	**Survey 1**	**Survey 2**	**Survey 3**
Parus major	0.698 (0.120)	0.701 (0.112)	0.890 (0.096)	0.700 (0.121)	0.703 (0.113)	0.892 (0.093)	0.701 (0.122)	0.704 (0.114)	0.892 (0.093)
Cyanistes caeruleus	0.793 (0.098)	0.720 (0.127)	0.791 (0.087)	0.795 (0.096)	0.722 (0.127)	0.793 (0.086)	0.795 (0.097)	0.722 (0.127)	0.793 (0.087)
Aegithalos caudatus	0.581 (0.125)	0.583 (0.122)	0.611 (0.142)	0.598 (0.121)	0.600 (0.117)	0.627 (0.135)	0.611 (0.136)	0.613 (0.133)	0.640 (0.146)
Sitta europaea	0.651 (0.272)	0.532 (0.309)	0.600 (0.253)	0.749 (0.238)	0.630 (0.319)	0.698 (0.238)	0.749 (0.238)	0.630 (0.319)	0.698 (0.238)
Certhia brachydactyla	0.340 (0.184)	0.344 (0.194)	0.345 (0.186)	0.329 (0.177)	0.332 (0.188)	0.334 (0.180)	0.322 (0.180)	0.326 (0.191)	0.327 (0.183)
Lanius senator	0.872 (0.334)	0.954 (0.158)	1.000 (0.000)	0.872 (0.334)	0.954 (0.158)	1.000 (0.000)	0.872 (0.334)	0.954 (0.158)	1.000 (0.000)
Garrulus glandarius	0.394 (0.128)	0.394 (0.128)	0.394 (0.128)	0.862 (0.085)	0.862 (0.085)	0.862 (0.085)	0.998 (0.022)	0.998 (0.022)	0.998 (0.022)
Pica pica	0.233 (0.365)	0.233 (0.365)	0.229 (0.359)	0.931 (0.137)	0.931 (0.137)	0.927 (0.140)	0.931 (0.137)	0.931 (0.137)	0.927 (0.140)
Petronia petronia	0.745 (0.226)	0.904 (0.113)	0.904 (0.113)	0.750 (0.224)	0.909 (0.101)	0.909 (0.101)	0.757 (0.227)	0.916 (0.098)	0.916 (0.098)
Fringilla coelebs	0.910 (0.057)	0.928 (0.043)	0.940 (0.046)	0.906 (0.058)	0.924 (0.046)	0.936 (0.050)	0.921 (0.061)	0.939 (0.044)	0.951 (0.044)
Serinus serinus	0.956 (0.040)	0.959 (0.040)	0.954 (0.043)	0.972 (0.026)	0.975 (0.025)	0.970 (0.031)	0.972 (0.026)	0.975 (0.025)	0.970 (0.031)
Carduelis chloris	0.929 (0.107)	0.959 (0.048)	0.959 (0.048)	0.923 (0.108)	0.954 (0.054)	0.954 (0.054)	0.929 (0.107)	0.959 (0.048)	0.959 (0.048)
Carduelis carduelis	0.824 (0.106)	0.772 (0.123)	0.837 (0.106)	0.847 (0.080)	0.794 (0.112)	0.859 (0.076)	0.854 (0.083)	0.801 (0.117)	0.866 (0.078)
Linaria cannabina	0.888 (0.114)	0.925 (0.125)	0.712 (0.143)	0.935 (0.058)	0.971 (0.050)	0.759 (0.165)	0.948 (0.058)	0.985 (0.039)	0.772 (0.179)
Loxia curvirostra	0.674 (0.108)	0.674 (0.108)	0.674 (0.108)	0.994 (0.030)	0.994 (0.030)	0.994 (0.031)	0.994 (0.030)	0.994 (0.030)	0.994 (0.031)
Emberiza calandra	0.532 (0.348)	0.695 (0.204)	0.567 (0.310)	0.527 (0.345)	0.690 (0.202)	0.562 (0.307)	0.525 (0.345)	0.688 (0.203)	0.561 (0.307)
Emberiza cia	0.616 (0.171)	0.735 (0.103)	0.981 (0.060)	0.618 (0.173)	0.736 (0.104)	0.983 (0.054)	0.618 (0.173)	0.737 (0.105)	0.983 (0.053)
Emberiza cirlus	0.776 (0.082)	0.778 (0.069)	0.781 (0.070)	0.780 (0.080)	0.782 (0.066)	0.785 (0.067)	0.778 (0.080)	0.781 (0.066)	0.784 (0.067)
